# Expression pattern of Ccr2 and Cx3cr1 in inherited retinal degeneration

**DOI:** 10.1186/s12974-015-0408-3

**Published:** 2015-10-12

**Authors:** Hideo Kohno, Hideto Koso, Kiichiro Okano, Thomas R. Sundermeier, Saburo Saito, Sumiko Watanabe, Hiroshi Tsuneoka, Tsutomu Sakai

**Affiliations:** Department of Ophthalmology, The Jikei University School of Medicine, 105-8461 Tokyo, Japan; Department of Molecular Immunology, The Jikei University School of Medicine, 105-8461 Tokyo, Japan; Tokyu Hospital, 145-0062 Tokyo, Japan; Division of Molecular and Developmental Biology, The Institute of Medical Science, The University of Tokyo, 108-8639 Tokyo, Japan; Department of Pharmacology, School of Medicine, Case Western Reserve University, Cleveland, OH USA

**Keywords:** Microglia, Chemokine, Photoreceptor, Cell migration, Inflammation

## Abstract

**Background:**

Though accumulating evidence suggests that microglia, resident macrophages in the retina, and bone marrow-derived macrophages can cause retinal inflammation which accelerates photoreceptor cell death, the details of how these cells are activated during retinal degeneration (RD) remain uncertain. Therefore, it is important to clarify which cells play a dominant role in fueling retinal inflammation. However, distinguishing between microglia and macrophages is difficult using conventional techniques such as cell markers (e.g., Iba-1). Recently, two mouse models for visualizing chemokine receptors were established, *Cx3cr1*^*GFP/GFP*^ and *Ccr2*^*RFP/RFP*^ mice. As *Cx3cr1* is expressed in microglia and *Ccr2* is reportedly expressed in activated macrophages, these mice have the potential to distinguish microglia and macrophages, yielding novel information about the activation of these inflammatory cells and their individual roles in retinal inflammation.

**Methods:**

In this study, *c-mer proto-oncogene tyrosine kinase* (*Mertk*)^*−/−*^ mice, which show photoreceptor cell death due to defective retinal pigment epithelium phagocytosis, were employed as an animal model of RD. *Mertk*^*−/−*^*Cx3cr1*^*GFP/+*^*Ccr2*^*RFP/+*^ mice were established by breeding *Mertk*^*−/−*^, *Cx3cr1*^*GFP/GFP*^, and *Ccr2*^*RFP/RFP*^ mice. The retinal morphology and pattern of inflammatory cell activation and invasion of *Mertk*^*−/−*^*Cx3cr1*^*GFP/+*^*Ccr2*^*RFP/+*^ mice were evaluated using retina and retinal pigment epithelium (RPE) flat mounts, retinal sections, and flow cytometry.

**Results:**

Four-week-old *Mertk*^*−/−*^*Cx3cr1*^*GFP/+*^*Ccr2*^*RFP/+*^ mice showed Cx3cr1-GFP-positive microglia in the inner retina. Cx3cr1-GFP and Ccr2-RFP dual positive activated microglia were observed in the outer retina and subretinal space of 6- and 8-week-old animals. Ccr2-RFP single positive bone marrow-derived macrophages were observed to migrate into the retina of *Mertk*^*−/−*^*Cx3cr1*^*GFP/+*^*Ccr2*^*RFP/+*^ mice. These invading cells were still observed in the subretinal space in 18-week-old animals.

**Conclusions:**

Cx3cr1-GFP-positive microglia and Ccr2-RFP-positive macrophages were distinguishable in the retinas of *Mertk*^*−/−*^*Cx3cr1*^*GFP/+*^*Ccr2*^*RFP/+*^ mice. In addition, Ccr2 expression in Cx3cr1 positive microglia is a feature of microglial activation in RD. *Mertk*^*−/−*^*Cx3cr1*^*GFP/+*^*Ccr2*^*RFP/+*^ mice enabled observation of microglial activation over time during RD and may be useful for developing inflammation-targeted treatment strategies for RD in the future.

## Background

Since photoreceptor cell death (PCD) is the proximal cause of blindness in retinal degenerative disorders such as retinitis pigmentosa and age-related macular degeneration, the mechanism of PCD should be clarified. Though traditionally PCD is thought to occur due to genetic predisposition, environmental risk factors, and old age [1], accumulating clinical and experimental evidence suggests that retinal inflammation can accelerate PCD [2–5]. Retinal inflammation is mediated by the retinal innate immune system, including microglia, which are resident macrophages in the central nervous system (CNS) and the complement system [1]. In the healthy retina, the retinal innate immune system plays a beneficial role in maintaining retinal homeostasis [1]. However, once a pro-inflammatory cascade is triggered, this retinal innate immune system can cause PCD [3]. The molecular details of the retinal inflammatory cascade, PCD, and the relationship between the two are not yet entirely understood. However, it is clear that not only microglia but also bone marrow-derived macrophages which invade to retina via a damaged blood-retina barrier play principal roles in this process [3].

We recently reported migration of microglia and macrophages into the subretinal space in during retinal degeneration (RD) [3]. As both of these cell types are stained by the Iba-1 Ab, it is unclear which is the dominant cell type in the subretinal space. To differentiate the contributions of microglia and macrophages in PCD, we administered the microglia suppressive drug “minocycline” or depleted macrophages systemically by injection of clodronate-liposomes in an RD animal model. However, interestingly, both of these treatments ameliorated PCD in light exposed *Abca4*^*−/−*^*Rdh8*^*−/−*^ mice which show drastic RD [3, 6], indicating that both microglia and macrophages play important roles in retinal inflammation and degeneration. Therefore, it is still uncertain which cell type initially triggers retinal inflammation and which plays a more dominant role in driving subsequent PCD.

In the current study, we employed two fluorescein protein knock in mouse models, namely *Cx3cr1*^*GFP/GFP*^ and *Ccr2*^*RFP/RFP*^ mice [7, 8] to distinguish microglia and macrophage in the retina. Cx3cr1 is the sole receptor for Cx3cl1, also called fractalkine. Cx3cr1 is expressed by dendritic cells, natural killer cells, and macrophages [9]. Ccr2 is also the sole receptor for Ccl2. Ccr2 is required for macrophage infiltration to injure cites [10]. Furthermore, both Cx3cr1 and Ccr2 are upregulated in RD [3, 11]. In a study of the brain, Cx3cr1 but not Ccr2 was expressed in microglia from embryonic development throughout adulthood [12]. However, whether this principle applies to retinal degeneration remains unknown. To shed light on microglia activation and to test whether microglia and macrophages are distinguishable in retinal degeneration, *c-mer proto-oncogene tyrosine kinase* (*Mertk*)^*−/−*^*Cx3cr1*^*GFP/+*^*Ccr2*^*RFP/+*^ or *Mertk*^*+/+*^*Cx3cr1*^*GFP/+*^*Ccr2*^*RFP/+*^ mice were established by breeding *Mertk*^*−/−*^, *Cx3cr1*^*GFP/GFP*^, and *Ccr2*^*RFP/RFP*^ mice. Mertk plays an essential role in retinal pigment epithelium (RPE) phagocytosis [13], and *Mertk* deficiency causes RD [14]. Furthermore, *Mertk*^*−/−*^ mice show retinal inflammation associated with microglia and macrophage accumulation in the subretinal space [3, 11]. The retinal morphology and expression pattern of Cx3cr1-GFP and Ccr2-RFP in *Mertk*^*−/−*^*Cx3cr1*^*GFP/+*^*Ccr2*^*RFP/+*^ mice was examined by retinal sectioning, retina and RPE flat mounts, and flow cytometry.

## Methods

### Animals

*Mertk*^*−/−*^, *Cx3cr1*^*GFP/GFP*^, and *Ccr2*^*RFP/RFP*^ mice were obtained from Jackson Lab (Bar Harbor, Maine). Genotyping for *Mertk* was performed with primers: for wild type, forward 5′-GCTTTAGCCTCCCCAGTAGC-3′, reverse 5′-GGTCACATGCAAAGCAAATG-3′ and for mutant, forward 5′-CGTGGAGAAGGTAGTCGTACATCT-3′ and reverse 5′-TTTGCCAAGTTCTAATTCCATC-3′. Genotyping was performed for *Cx3cr1* with primers; for wild type, forward 5′-TCCACGTTCGGTCTGGTGGG-3′ and reverse 5′-GGTTCCTAGTGGAGCTAGGG-3′ and for *Cx3cr1* mutant, forward 5′-GATCACTCTCGGCATGGACG-3′ and reverse 5′-GGTTCCTAGTGGAGCTAGGG-3′. Genotyping for *Ccr2* was performed with primers: for common, forward 5′-TAAACCTGGTCACCACATGC-3′; for wild type, reverse 5′-GGAGTAGAGTGGAGGCAGGA-3′; and for *Ccr2* mutant, reverse 5′-CTTGATGACGTCCTCGGAG-3′, according to the protocol from Jackson Lab. *Mertk*^*−/−*^ mice were crossed with C57BL/6 mice to make *Mertk*^*+/−*^ mice. *Mertk*^*−/−*^ and littermate control (*Mertk*^*+/+*^) mice, which were used as WT mice, were derived from *Mertk*^*+/−*^ parents. *Mertk*^*−/−*^*Cx3cr1*^*GFP/+*^*Ccr2*^*RFP/+*^ and *Cx3cr1*^*GFP/+*^*Ccr2*^*RFP/+*^ mice were established from the same mouse lines. Briefly, since Cx3cr1 and Ccr2 are on the same mouse chromosome (Chr 9), *Mertk*^*+/−*^*Cx3cr1*^*GFP/+*^ and *Mertk*^*+/−*^*Ccr2*^*RFP/+*^ mice were established first from breeding *Mertk*^*−/−*^ mice with *Cx3cr1*^*GFP/GFP*^ mice or *Mertk*^*−/−*^ mice with *Ccr2*^*RFP/RFP*^ mice, respectively. *Mertk*^*−/−*^*Cx3cr1*^*GFP/GFP*^ and *Cx3cr1*^*GFP/GFP*^ (littermate control of *Mertk*^*−/−*^*Cx3cr1*^*GFP/GFP*^) were obtained from *Mertk*^*+/−*^*Cx3cr1*^*GFP/+*^ parents. *Mertk*^*−/−*^*Ccr2*^*RFP/RFP*^ and *Ccr2*^*RFP/RFP*^ (littermate control of *Mertk*^*−/−*^*Ccr2*^*RFP/RFP*^) were obtained from *Mertk*^*+/−*^*Ccr2*^*RFP/+*^ parents. *Mertk*^*−/−*^*Cx3cr1*^*GFP/+*^*Ccr2*^*RFP/+*^ mice were established by breeding *Mertk*^*−/−*^*Cx3cr1*^*GFP/GFP*^ and *Mertk*^*−/−*^*Ccr2*^*RFP/RFP*^ mice. *Cx3cr1*^*GFP/+*^*Ccr2*^*RFP/+*^ mice were established by breeding *Cx3cr1*^*GFP/GFP*^ (littermate control of *Mertk*^*−/−*^*Cx3cr1*^*GFP/GFP*^) and *Ccr2*^*RFP/RFP*^ (littermate control of *Mertk*^*−/−*^*Ccr2*^*RFP/RFP*^).

Equal numbers of males and females were used. All mice were housed in the animal facility at the Jikei University School of Medicine, where they were maintained either under complete darkness or on a 12-h light (~10 lux)/12-h dark cycle. All animal procedures and experiments were approved by the Jikei University School of Medicine Animal Care Committees and conformed to both the recommendations of the American Veterinary Medical Association Panel on Euthanasia and the Association of Research for Vision and Ophthalmology.

### Flat mount retina and RPE preparation

All procedures for retina and RPE flat mounts were described previously [3]. Images of flat mounts were captured by a confocal microscope (LSM, Carl Zeiss, Thornwood, NY, USA). For retina flat mount, the entire retina was captured at 5 μm intervals and all photographs were projected in one slice. For RPE flat mounts, the entire visible RPE was captured at 3 μm intervals and projected in one slice.

### Histological analysis

All procedures to make sections for light microscopy were performed using a previously described method [15]. Rabbit anti-Iba-1 Ab (1:400, Wako, Osaka, Japan) was used for immunohistochemistry (IHC). Cell number was counted using ImageJ (National Institutes of Health, Bethesda, MD, USA). To observe microglia cell bodies, low melting point agarose-embedded (Sigma, St. Louis, MO, USA) thick sections (100 μm thickness) were prepared [16]. Images of IHC were captured on a confocal microscope (LSM, Carl Zeiss, Thornwood, NY, USA).

### Quantitative RT-PCR (qRT-PCR)

All procedures for qRT-PCR were described previously [3]. Briefly, retinal samples from each group were collected from 16 eyes. Total RNA was isolated using a RiboPure Kit (Applied Biosystems, Austin, TX, USA), and cDNA was synthesized with SuperScript™ II Reserve Transcriptase (Invitrogen) following the manufacturer’s instructions. Real-time PCR amplification was performed using iQ™ SYBR II Green Supermix (Bio-Rad). Primers were designed using the web tool Primer 3 and synthesized by Eurofins MWG Operon (Hunstville, AL, USA). The following primers were used for analyses: *Cx3cr1* (202 bp), forward 5′-CACCATTAGCTGGGCGTCT-3′, reverse 5′-GATGCGGAAGTAGCAAAAGC-3′; *Ccr2* (227 bp), forward 5′-ATTCTCCACACCCTGTTTCG-3′, reverse 5′-ATGCAGCAGTGTGTCATTCC-3′. Relative expression of genes was normalized by comparison to the housekeeping gene *Gapdh*.

### Flow cytometry

The neural retina was isolated from the eyecup with minimal inclusion of RPE cells, and the choroid plexus and ciliary body were carefully removed. The retina was then incubated in 0.25 % trypsin/PBS at 37 °C for 15 min. After stopping the activity of trypsin by the addition of 10 % FBS/PBS with 0.1 % DNaseI (Invitrogen, Waitham, MA, USA), the cells were mechanically dissociated into a single-cell suspension by gentle pipetting. The dissociated cells were stained with 0.1 % propidium iodide (PI)/PBS, and 100,000 cells were analyzed by FACSCalibur (BD, Franklin Lakes, NJ, USA). Peripheral blood samples were collected from the orbital venous plexus. After the lysis of erythrocytes in an isotonic solution of ammonium chloride, white blood cells (WBC) were stained with PI and used for flow cytometric analysis. Data analysis was performed using FlowJo software.

### Data analysis

Data represent the mean ± SD. At least three independent experiments were compared by the one-way analysis of variance test.

## Results

First, the retinal phenotype of *Ccr2*^*RFP/RFP*^ and *Cx3cr1*^*GFP/GFP*^ mice was analyzed. No Ccr2-RFP-positive cells were detected in the retina of *Ccr2*^*RFP/RFP*^ mice, but Cx3cr1-GFP-positive microglia were ubiquitous in the inner retina (from the ganglion cell layer to the inner nuclear layer) of *Cx3cr1*^*GFP/GFP*^ mice. Ccr2-RFP-positive cells were found in *Ccr2*^*RFP/RFP*^ mice in the blood cell fraction corresponding to the monocyte population. No retinal degeneration was observed in either *Ccr2*^*RFP/RFP*^ or *Cx3cr1*^*GFP/GFP*^ mice (data not shown).

### Retinal degeneration, microglia migration, and increase of Cx3cr1 and Ccr2 in Mertk^−/−^ mice

**Fig. 1:**
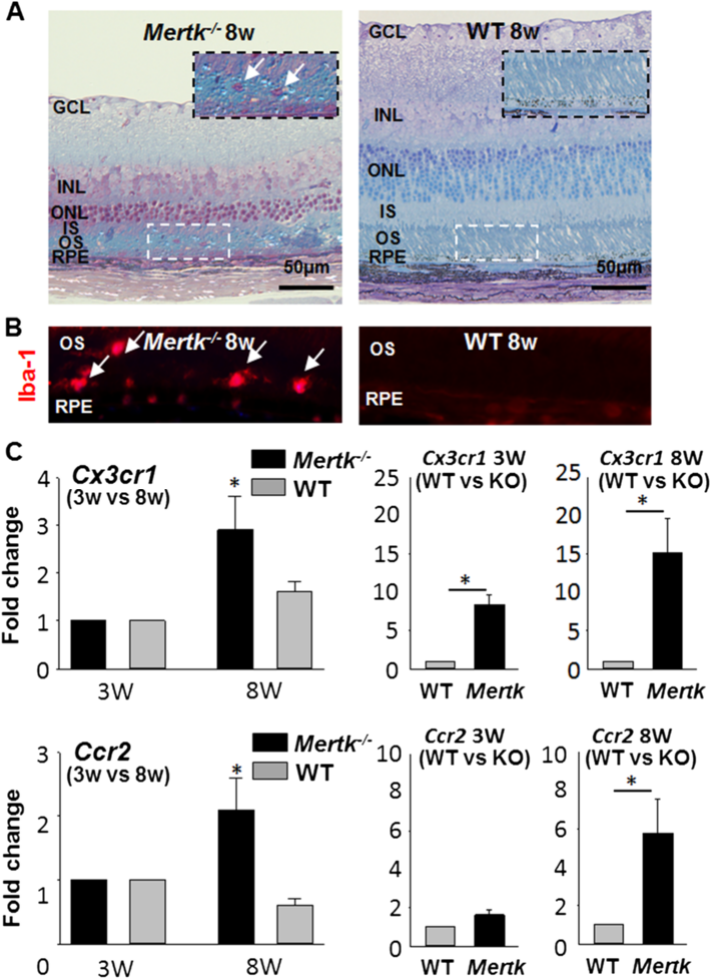
Retinal phenotype and increase of *Cx3cr1* and *Ccr2* mRNA levels in *Mertk* ^*−/−*^ mice. *Mertk* ^*−/−*^ or WT (*Mertk* ^*+/+*^) mice were established from *Mertk* ^*+/−*^ mice. **a** Retinal sections of 8-week-old *Mertk* ^*−/−*^ (*left*) and WT (*right*) mice were prepared. Eight-week-old *Mertk* ^*−/−*^mice developed retinal degeneration, represented by thinning of the outer nuclear layer (*ONL*) and migration of inflammatory cells (*arrow*) (*left panel*). The *insets* are magnified images of the area within the *broken rectangle. GCL* ganglion cell layer, *INL* inner nuclear layer, *ONL* outer nuclear layer, *IS* inner segments, *OS* outer segments, *RPE* retinal pigment epithelium. **b** IHC was performed using rabbit anti-Iba-1-Ab. Eight-week-old *Mertk* ^*−/−*^ mice showed Iba-1-positive microglia/macrophages in the outer retina, though no Iba-1-positive cells were observed in WT mice. **c** *Cx3cr1* and *Ccr2* mRNA levels in the retina of *Mertk* ^*−/−*^ and WT were measured by qPCR. RNA samples were collected from 16 retinas at each time point. qPCR was performed 3–6 times (*n* = 3–6). Expression levels were compared between 3- and 8-week-old animals and between WT and *Mertk* ^*−/−*^ mice at each age. *Error bars* indicate the SD of the mean (*n* > 3). *Asterisk* indicates *P* < 0.05 vs 3-week-old *Mertk* ^*−/−*^ mice

Next, the retinal phenotype of *Mertk*^*−/−*^ and WT (*Mertk*^*+/+*^; littermate control of *Mertk*^*−/−*^) mice was evaluated. Eight-week-old *Mertk*^*−/−*^ mice showed a decrease in photoreceptor nuclei number in the outer nuclear layer (ONL) and disorganized inner segments (IS) and outer segments (OS) (Fig. 1a). Iba-1-positive cells were found to migrate to the IS, OS, and subretinal space in 8-week-old *Mertk*^*−/−*^ mice (Fig. 1b) indicating microglia/macrophage migration [3]. Because Iba-1 is expressed in both microglia and macrophages, these cells were not distinguishable. qPCR was used to compare *Cx3cr1* and *Ccr2* mRNA levels in the retinas of 3- and 8-week-old *Mertk*^*−/−*^ and WT (Fig 1c). No significant differences in *Cx3cr1* or *Ccr2* expression were observed between 3- and 8-week-old WT mice. In contrast, for *Mertk*^*−/−*^ mice, these mRNAs were increased in 8-week-old mice as compared to 3-week-old animals (Fig. 1c, left). At both the 3- and 8-week time points, expression of *Cx3cr1* was increased in *Mertk*^*−/−*^ mice compared to WT mice, and *Ccr2* levels were increased in 8-week-old *Mertk*^*−/−*^ mice as compared to WT controls (Fig. 1c, right).

### Migration of Cx3cr1-GFP-expressing microglia and invasion of Ccr2-RFP-positive monocyte-derived macrophage in degenerating retinas

**Fig. 2:**
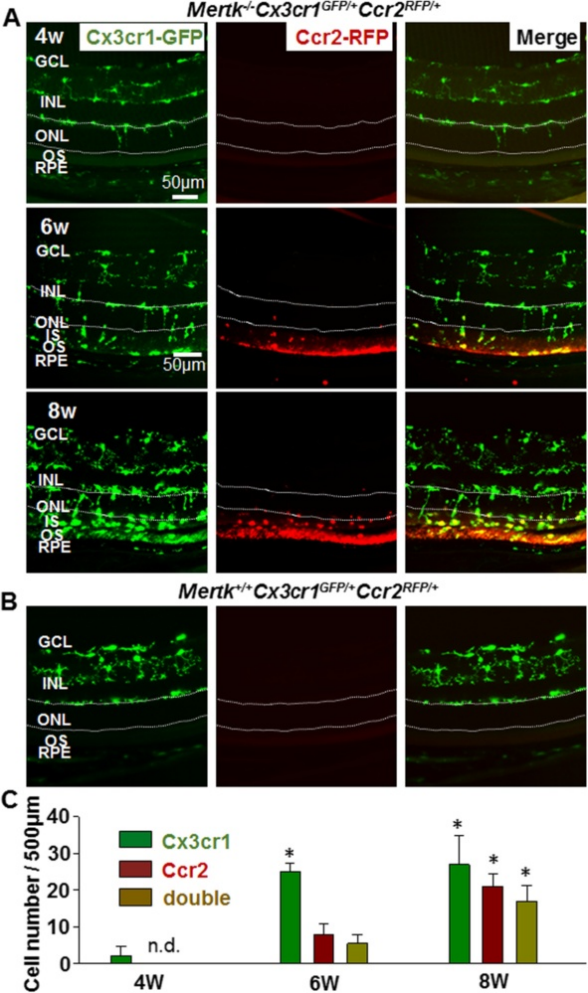
Cx3cr1-GFP-positive microglia and Ccr2-RFP-positive macrophages in the retinas of *Mertk* ^*−/−*^ *Cx3cr1* ^*GFP/+*^ *Ccr2* ^*RFP/+*^ mice. **a** Retinal sections of *Mertk* ^*−/−*^ *Cx3cr1* ^*GFP/+*^ *Ccr2* ^*RFP/+*^ mice at 4 (*upper panels*), 6 (middle panels), and 8 weeks of age (*lower panels*) are shown. Cx3cr1-GFP is shown in *green*, and Ccr2-RFP is shown in *red. GCL* ganglion cell layer, *INL* inner nuclear layer, *ONL* outer nuclear layer, *IS* inner segments, *OS* outer segments, *RPE* retinal pigment epithelium. The ONL boundaries are marked with *broken lines*. **b** Retinal sections of 8-week-old *Cx3cr1* ^*GFP/+*^ *Ccr2* ^*RFP/+*^ mice are shown as a negative control. **c** The numbers of Cx3cr1-GFP, Ccr2-RFP, and Cx3cr1-GFP and Ccr2-RFP dual positive cells in 500 μm of the outer retina (from ONL to OS), and RPE of *Mertk* ^*−/−*^ *Cx3cr1* ^*GFP/+*^ *Ccr2* ^*RFP/+*^ mice were counted. Five different sections from different mice were used to count cell number. *Asterisk* indicates *P* < 0.05 vs 4-week-old *Mertk* ^*−/−*^ *Cx3cr1* ^*GFP/+*^ *Ccr2* ^*RFP/+*^ mice. *n.d.* not detected

To test the expression pattern of *Ccr2* and *Cx3cr1* in the degenerating retina and to determine whether microglia and macrophages are distinguishable in RD using *Ccr2*^*RFP/RFP*^ and *Cx3cr1*^*GFP/GFP*^ mice (as previously reported in the brain [12]), *Mertk*^*−/−*^*Cx3cr1*^*GFP/+*^*Ccr2*^*RFP/+*^ mice were established. Retinal sections of 4-, 6-, and 8-week-old *Mertk*^*−/−*^*Cx3cr1*^*GFP/+*^*Ccr2*^*RFP/+*^ mice are shown in Fig. 2. Four-week-old *Mertk*^*−/−*^*Cx3cr1*^*GFP/+*^*Ccr2*^*RFP/+*^ mice, at the onset of RD, showed only Cx3cr1-GFP-positive microglia in the retina, and migration of some of these cells to the ONL was observed. No Ccr2-RFP-positive macrophages were observed (Fig. 2a, upper panels). Breakdown of the blood-retina barrier during the progression of retinal degeneration in *Mertk*^*−/−*^ mice was previously reported [11]. Six-week-old *Mertk*^*−/−*^*Cx3cr1*^*GFP/+*^*Ccr2*^*RFP/+*^ mice showed not only Cx3cr1-GFP-positive microglia but also Ccr2-RFP-positive macrophages in the outer retina, including the ONL, IS, OS, and RPE layers (Fig. 2a, middle panels). Interestingly, some RPE cells also expressed Ccr2-RFP. At 8 week of age, abundant Cx3cr1-GFP-positive and Ccr2-RFP-positive cells were observed. The majority of invading cells in the subretinal space (between the OS and RPE layers) were Cx3cr1-GFP and Ccr2-RFP dual positive (Fig. 2a, lower panels). Retinal sections of *Cx3cr1*^*GFP/+*^*Ccr2*^*RFP/+*^ 8-week-old mice at are shown as negative control (Fig. 2b). Cell numbers in the outer retina (from ONL to OS) and RPE were counted (Fig. 2c). Ccr2-RFP-positive and Cx3cr1-GFP and Ccr2-RFP dual positive cells increased in 8-week-old *Mertk*^*−/−*^*Cx3cr1*^*GFP/+*^*Ccr2*^*RFP/+*^ mice (Fig. 2a, lower panels, and Fig. 2c).

### Microglia that have migrated to the subretinal space are Cx3cr1-GFP and Ccr2-RFP double positive

**Fig. 3:**
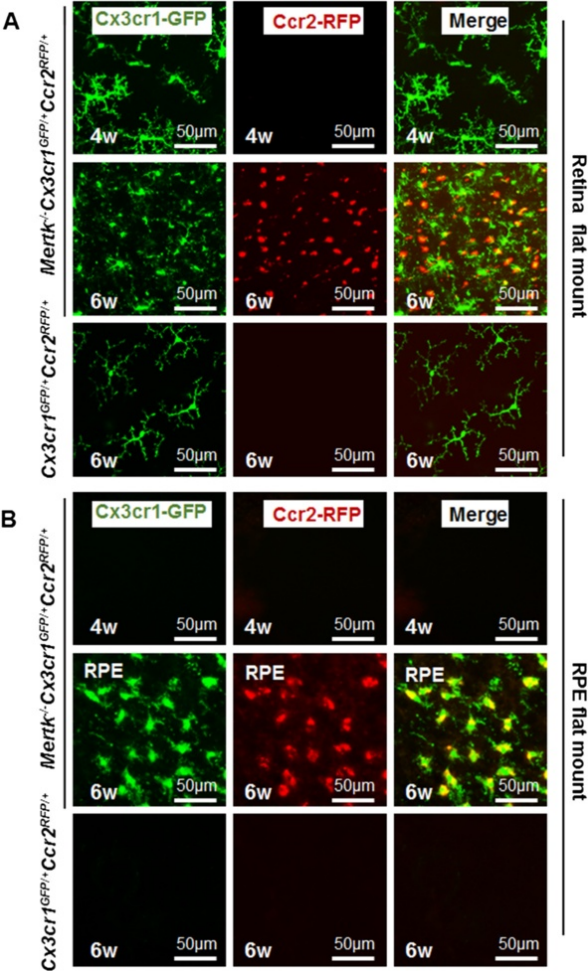
Retina and RPE flat mounts from *Mertk* ^*−/−*^ *Cx3cr1* ^*GFP/+*^ *Ccr2* ^*RFP/+*^ mice. Retina and RPE flat mounts from *Mertk* ^*−/−*^ *Cx3cr1* ^*GFP/+*^ *Ccr2* ^*RFP/+*^ mice were prepared to analyze Cx3cr1-GFP and Ccr2-RFP-positive cells in more detail. **a** Retina flat mounts from 4- (*upper panels*) and 6-week-old (*middle panels*) *Mertk* ^*−/−*^ *Cx3cr1* ^*GFP/+*^ *Ccr2* ^*RFP/+*^ mice are shown. Retina flat mounts from 6-week-old *Cx3cr1* ^*GFP/+*^ *Ccr2* ^*RFP/+*^ mice (*lower panels*) are shown as negative controls. Cx3cr1-GFP is shown in *green,* and Ccr2-RFP is shown in *red*. **b** RPE flat mounts from 4- (*upper panels*) and 6-week-old (*middle panels*) *Mertk* ^*−/−*^ *Cx3cr1* ^*GFP/+*^ *Ccr2* ^*RFP/+*^ mice are shown. RPE flat mounts from 6-week-old *Cx3cr1* ^*GFP/+*^ *Ccr2* ^*RFP/+*^ mice (*lower panels*) are shown as negative controls. Cx3cr1-GFP is shown in *green,* and Ccr2-RFP is shown in *red*

**Fig. 4:**
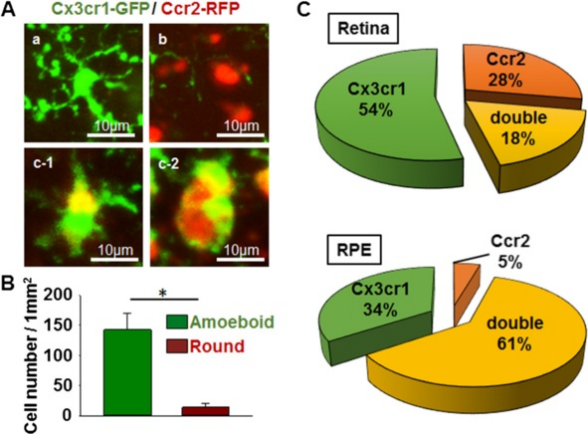
Analysis of retina and RPE flat mount. **a** Representative Cx3cr1-GFP (*a*), Ccr2-RFP (*b*), and Cx3cr1-GFP and Ccr2-RFP dual positive cells (amoeboid shape (*c-1*) and round shape (*c-2*)) observed in the retina and RPE flat mounts of 6-week-old *Mertk* ^*−/−*^ *Cx3cr1* ^*GFP/+*^ *Ccr2* ^*RFP/+*^ mice are shown. Cx3cr1-GFP and Ccr2-RFP merged images are shown. **b**, **c** The number of amoeboid- (*a*-*c-1*) or round-shaped (*a*-*c-2*) Cx3cr1-GFP and Ccr2-RFP dual positive cells observed in RPE flat mounts (1 mm^2^) of 6-week-old *Mertk* ^*−/−*^ *Cx3cr1* ^*GFP/+*^ *Ccr2* ^*RFP/+*^ mice was counted. Five different RPE flat mounts from different mice were used to count cell number. *Asterisk* indicates *P* < 0.05. **b** Bar graph shows the abundance of amoeboid- and round-shaped dual positive cells in RPE flat mounts. **c** Pie chart displays the ratios of Cx3cr1-GFP-positive, Ccr2-RFP-positive, and dual positive cells in the retina (*upper*) and RPE (*lower*)

Retinal and RPE flat mounts of *Mertk*^*−/−*^*Cx3cr1*^*GFP/+*^*Ccr2*^*RFP/+*^ mice were prepared to examine Cx3cr1-GFP-positive and Ccr2-RFP-positive cells in more detail (Fig. 3). Retinal and RPE flat mounts of *Cx3cr1*^*GFP/+*^*Ccr2*^*RFP/+*^ mice are shown as negative controls (Fig. 3a, b, lower panels). At 6 weeks, *Mertk*^*−/−*^*Cx3cr1*^*GFP/+*^*Ccr2*^*RFP/+*^ mice showed Cx3cr1-GFP-positive cells in the retina, which showed a ramified or amoeboid shape, corresponding to microglia. In addition, Ccr2-RFP-positive cells, which showed a round shape corresponding to monocyte-derived macrophages, were observed at this age (Fig. 3a, middle panels; Fig. 4a (a) and (b)). In contrast, only Cx3cr1-GFP-positive ramified-shaped microglia were observed in retina flat mounts of 4-week-old *Mertk*^*−/−*^*Cx3cr1*^*GFP/+*^*Ccr2*^*RFP/+*^ mice (Fig. 3a (a), upper panels). RPE flat mounts of 6-week-old *Mertk*^*−/−*^*Cx3cr1*^*GFP/+*^*Ccr2*^*RFP/+*^ mice showed amoeboid-shaped Cx3cr1-GFP-positive cells which co-express Ccr2-RFP, indicating that the microglia that had migrated to the subretinal space (the area just above the RPE) were Cx3cr1 and Ccr2 dual positive (Fig. 3b, middle panels, and Fig. 4a (c-1)). These Cx3cr1-GFP and Ccr2-RFP dual positive cells were morphologically distinct from Cx3cr1-GFP single positive ramified resting microglia and Ccr2-RFP single positive round-shaped macrophages (Fig. 4a). Amoeboid-shaped cells (Fig. 4a (c-1)), rather than round-shaped cells (Fig. 4a (c-2)), were the major cell population observed in RPE flat mounts from 6-week-old *Mertk*^*−/−*^*Cx3cr1*^*GFP/+*^*Ccr2*^*RFP/+*^ mice, indicating that microglia are the dominant inflammatory cell type in the subretinal space at 6 weeks of age. The cell number of amoeboid Cx3cr1-GFP and Ccr2-RFP dual positive cells is significantly higher than round-shaped Cx3cr1-GFP and Ccr2-RFP dual positive cells in RPE flat mounts of 6-week-old *Mertk*^*−/−*^*Cx3cr1*^*GFP/+*^*Ccr2*^*RFP/+*^ mice (Fig. 4b). Neither Cx3cr1-GFP-positive nor Ccr2-RFP-positive cells were observed in RPE flat mounts from 4-week-old *Mertk*^*−/−*^*Cx3cr1*^*GFP/+*^*Ccr2*^*RFP/+*^ mice (Fig. 3b, upper panels). Cx3cr1-GFP-positive and Ccr2-RFP-positive cells were counted. In 6-week-old *Mertk*^*−/−*^*Cx3cr1*^*GFP/+*^*Ccr2*^*RFP/+*^ mice, 18 and 61 % of the cells observed were Cx3cr1-GFP and Ccr2-RFP double positive in the retina and RPE, respectively (Fig. 4c). Taken together, Cx3cr1-GFP-positive microglia and Ccr2-RFP-positive macrophages were distinguishable in the sensory retina. However, microglia and macrophages that had migrated to the subretinal space, where accumulated OS and dying photoreceptors reside [3], co-expressed Cx3cr1-GFP and Ccr2-RFP, presumably indicating that activated microglia and macrophages that are actively phagocytizing photoreceptor OS express both of these factors.

### Age dependence of Cx3cr1-GFP and Ccr2-RFP-positive cell localization in the retina and RPE in Mertk^−/−^Cx3cr1^GFP/+^Ccr2^RFP/+^ mice

**Fig. 5:**
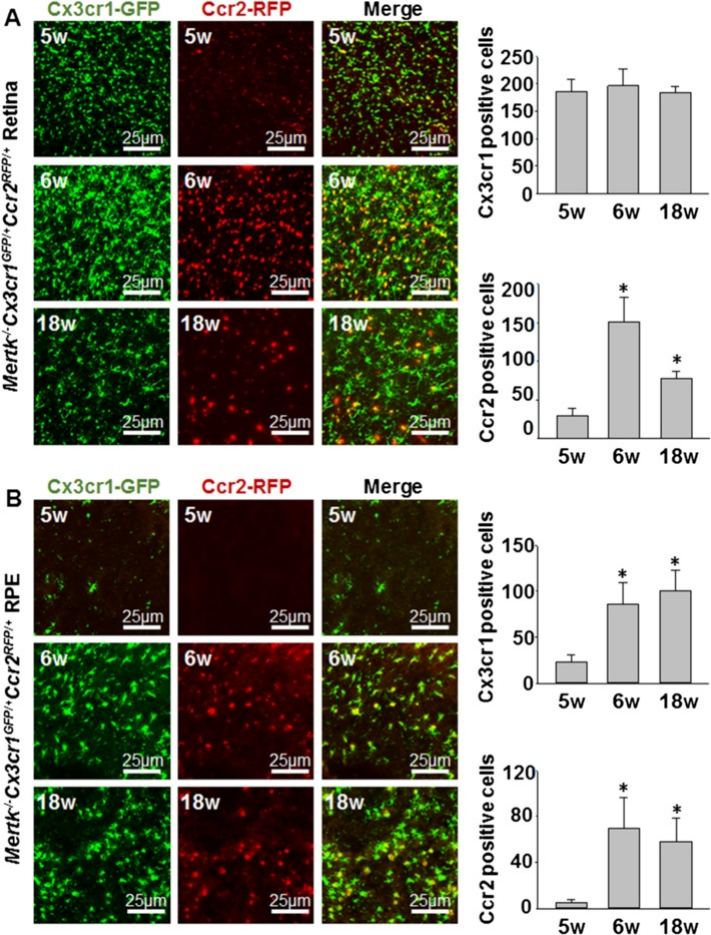
Retina and RPE flat mounts from *Mertk* ^*−/−*^ *Cx3cr1* ^*GFP/+*^ *Ccr2* ^*RFP/+*^ mice of different ages. **a** Retina flat mounts from 5-, 6-, and 18-week-old *Mertk* ^*−/−*^ *Cx3cr1* ^*GFP/+*^ *Ccr2* ^*RFP/+*^ mice are shown. The number of Cx3cr1-GFP and Ccr2-RFP-positive cells in retina flat mounts of 5-, 6-, and 18-week-old *Mertk* ^*−/−*^ *Cx3cr1* ^*GFP/+*^ *Ccr2* ^*RFP/+*^ mice was counted. **b** RPE flat mounts of 5-, 6-, and 18-week-old *Mertk* ^*−/−*^ *Cx3cr1* ^*GFP/+*^ *Ccr2* ^*RFP/+*^ mice are shown. The number of Cx3cr1-GFP and Ccr2-RFP-positive cells in RPE flat mounts of 5-, 6-, and 18-week-old *Mertk* ^*−/−*^ *Cx3cr1* ^*GFP/+*^ *Ccr2* ^*RFP/+*^ mice was counted. *Asterisk* indicates *P* < 0.05 vs 4-week-old *Mertk* ^*−/−*^ *Cx3cr1* ^*GFP/+*^ *Ccr2* ^*RFP/+*^ mice

Retina and RPE flat mounts from 5-, 6-, and 18-week-old *Mertk*^*−/−*^*Cx3cr1*^*GFP/+*^*Ccr2*^*RFP/+*^ mice are shown (Fig. 5). In retina flat mounts, the number of Ccr2-RFP-positive cells peaked at 6 weeks of age and slightly decreased in 18-week-old animals, though no significant difference in the number of Cx3cr1-GFP-positive cells was observed from 5 to 18 weeks (Fig. 5a). In RPE flat mounts, a small number of Cx3cr1-GFP-positive cells were observed at 5 weeks, and this number progressively increased in 6- and 18-week-old mice. A small number of Ccr2-RFP-positive cells were observed in the RPE at 5 weeks. Similar to the retina, this number peaked at 6 weeks and decreased slightly at 18 weeks (Fig. 5b).

### Flow cytometric analysis of Cx3cr1-GFP and Ccr2-RFP expression in white blood cells (WBC) and the retina

**Fig. 6:**
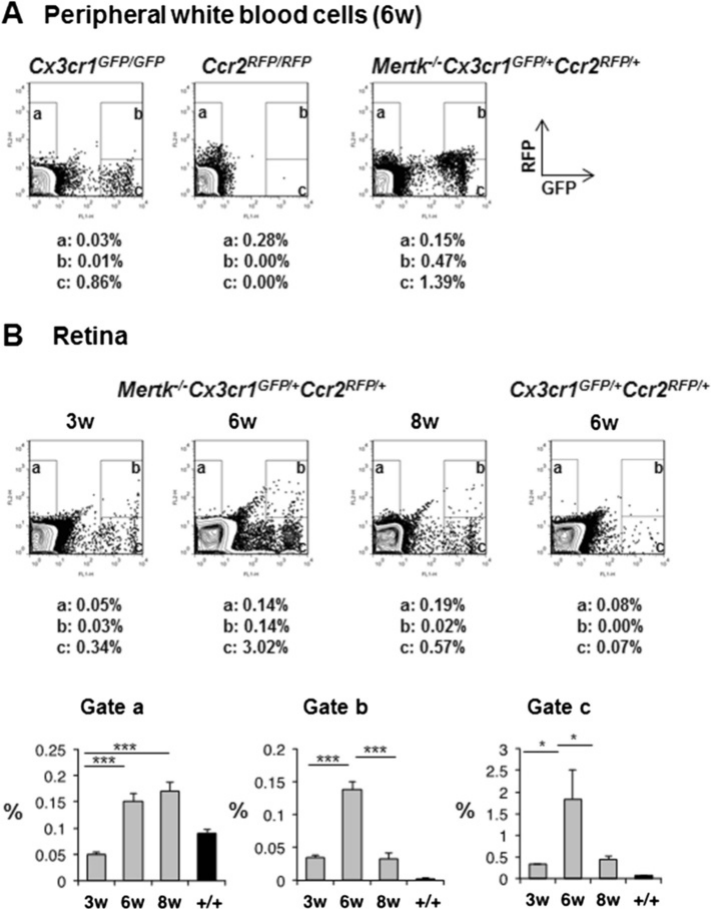
Flow cytometric analyses of WBC from *Cx3cr1* ^*GFP/GFP*^, *Ccr2* ^*RFP/RFP*^, and *Mertk* ^*−/−*^ *Cx3cr1* ^*GFP/+*^ *Ccr2* ^*RFP/+*^ mice and of the retinas from *Mertk* ^*−/−*^ *Cx3cr1* ^*GFP/+*^ *Ccr2* ^*RFP/+*^ mice. **a** WBC were harvested from *Cx3cr1* ^*GFP/GFP*^, *Ccr2* ^*RFP/RFP*^, and *Mertk* ^*−/−*^ *Cx3cr1* ^*GFP/+*^ *Ccr2* ^*RFP/+*^ mice. Cx3cr1-GFP and Ccr2-RFP are presented on the *X* and *Y axes*, respectively. Representative results from multiple flow cytometry analyses are shown. Boxes (*a*), (*b*), and (*c*) correspond to Ccr2-RFP, Ccr2-RFP, and Cx3cr1-GFP dual, and Cx3cr1-GFP-positive cells, respectively. **b** Neural retinas were obtained from 3-, 6-, and 8-week-old *Mertk* ^*−/−*^ *Cx3cr1* ^*GFP/+*^ *Ccr2* ^*RFP/+*^ mice and 6-week-old *Cx3cr1* ^*GFP/+*^ *Ccr2* ^*RFP/+*^ mice. Cx3cr1-GFP and Ccr2-RFP are presented on the *X* and *Y axes*, respectively. Representative results from multiple flow cytometry analyses are shown. Boxes (*a*), (*b*), and (*c*) correspond to Ccr2-RFP, Ccr2-RFP, and Cx3cr1-GFP dual, and Cx3cr1-GFP-positive cells, respectively. Quantitative results are shown for each gate. *Single asterisk* indicates *P* < 0.05, *double asterisks* indicate *P* < 0.005, and *triple asterisks* indicate *P* < 0.001 vs 3-week-old *Mertk* ^*−/−*^ *Cx3cr1* ^*GFP/+*^ *Ccr2* ^*RFP/+*^ mice. +/+ indicates 8-week-old *Cx3cr1* ^*GFP/+*^ *Ccr2* ^*RFP/+*^ mice

Since bone marrow-derived macrophages infiltrate the injured retina [17], circulating WBC in *Cx3cr1*^*GFP/GFP*^, *Ccr2*^*RFP/RFP*^, and *Mertk*^*−/−*^*Cx3cr1*^*GFP/+*^*Ccr2*^*RFP/+*^ mice were examined. The proportion of Cx3cr1-GFP and Ccr2-RFP cells in WBC from *Cx3cr1*^*GFP/GFP*^, *Ccr2*^*RFP/RFP*^, and *Mertk*^*−/−*^*Cx3cr1*^*GFP/+*^*Ccr2*^*RFP/+*^ mice was analyzed by flow cytometry. GFP- and RFP-positive WBC were observed in *Cx3cr1*^*GFP/GFP*^ and *Ccr2*^*RFP/RFP*^ mice, respectively (Fig. 6a, left and middle panels). WBC from *Mertk*^*−/−*^*Cx3cr1*^*GFP/+*^*Ccr2*^*RFP/+*^ mice showed the presence of Cx3cr1-GFP single positive cells (1.39 %), Ccr2-RFP single positive cells (0.15 %), and Cx3cr1-GFP and Ccr2-RFP dual positive cells (0.47 %) (Fig. 6a, right panel). The proportion of Cx3cr1-GFP and Ccr2-RFP cells in the retinas of *Mertk*^*−/−*^*Cx3cr1*^*GFP/+*^*Ccr2*^*RFP/+*^ mice was examined. The fraction of Ccr2-RFP-positive cells was increased in 6- and 8-week-old *Mertk*^*−/−*^*Cx3cr1*^*GFP/+*^*Ccr2*^*RFP/+*^ mice compared to 3-week-old animals (Fig. 6b, upper panels). The fraction of Cx3cr1-GFP-positive cells and Cx3cr1-GFP and Ccr2-RFP dual positive cells peaked at 6 weeks and returned to basal levels at 8 weeks (Fig. 6b, lower graphs). The proportion of Cx3cr1-GFP and Ccr2-RFP-positive cells in the retina of 6-week-old *Cx3cr1*^*GFP/+*^*Ccr2*^*RFP/+*^ mice is shown as a negative control.

## Discussion

Mutations in the *MERTK* gene cause retinal dystrophies in humans and in animal models [18]. MERTK belongs to a family of receptor tyrosine kinases that includes AXL and TYRO3 and plays an indispensable role in the clearance of photoreceptor debris by RPE phagocytosis [19]. Accumulation of photoreceptor debris in the subretinal space due to RPE phagocytosis deficiency is closely associated with the photoreceptor cell death seen in Royal College of Surgeons (RCS) rats (with disabled Mertk) and in *Mertk*^*−/−*^ mice [14]. Recently, we reported that *Mertk*^*−/−*^ mice show migration of microglia and retinal inflammation, which exacerbated retinal degeneration [3, 11]. Chemokines and cytokines including Ccl2, Ccl3, Ccl12, and Il1b are increased in the degenerating retinas of *Mertk*^*−/−*^*mice* [3, 11]. Furthermore, blockade of Ccl2 and Ccl3 can attenuate the retinal phenotype of *Mertk*^*−/−*^*mice*, clearly indicating that retinal inflammation contributes to PCD and RD [11].

To visualize inflammatory cells in a tissue, staining or immunohistochemistry procedures are required. However, tissue staining is limited for detecting inflammatory cells and immunohistochemistry requires clean and selective antibodies. Cx3cr1 and Ccr2, especially Ccr2, are difficult to detect by immunohistochemistry due to the lack of an appropriate antibody [7]. Newly developed fluorescent protein knock-in mouse models, including *Cx3cr1*^*GFP/GFP*^ and *Ccr2*^*RFP/RFP*^ mice have the potential to overcome the limitations of tissue staining and immunohistochemistry, and these mice will be instrumental for developing new treatment strategies, especially neuroinflammation-targeted therapy. From our current data, Cx3cr1-GFP and Ccr2-RFP dual positive cells were visualized not only by histology (Figs. 2 and 3) but also flow cytometry (Fig. 6).

To date, several studies used a combination of *Cx3cr1*^*GFP/GFP*^ and *Ccr2*^*RFP/RFP*^ mice in experimentally induced disease models such as experimental autoimmune encephalomyelitis (EAE), an animal model of multiple sclerosis [7, 12]. However, these mouse models have yet to be employed in naturally occurring neurodegenerative disease models such as retinal degeneration or Alzheimer’s disease. In experimentally induced disease models such as EAE, inflamed monocyte-derived macrophages play a dominant role in neuroinflammation and degeneration; hence, disease onset occurs outside the CNS. In contrast, in naturally occurring neurodegenerative diseases including retinal degeneration, microglia presumably play the dominant role at the onset of neuroinflammation and degeneration because the blood-retina barrier or blood-brain barrier is maintained at early disease stages [3, 11, 20]. This study provides evidence that microglia is the dominant inflammatory cell during the early stages of retinal degeneration (Figs. 2, 3, and 4). The precise mechanisms underlying microglial activation and morphological alteration during RD still remain unclear. However, evidence suggests that exposure to dead photoreceptor debris and subsequent phagocytosis is an important trigger for microglial activation in RD, as administration of photoreceptor OS proteins induced increased cytokine and chemokine production in microglia in vitro [3]. Currently, it remains unclear how Ccr2-RFP-positive macrophages infiltrate the retina. However, they likely invade via either the inner or outer blood-retina barrier. The inner blood-retina barrier is composed of tight junctions between neighboring capillary endothelial cells which rest on a basal lamina covered by the foot processes of astrocytes and Müller glia and tight junctions between RPE cells comprise the outer blood-retina barrier [21]. We previously reported disruption of the inner blood-retina barrier during RD [3], and the tight junctions between RPE cells are also reportedly damaged during this process [11, 22].

Ccr2 is a therapeutic target for retinal degenerative disorders including AMD and RP because deletion of Ccl2, a cognate ligand for Ccr2, can rescue PCD in *Mertk*^*−/−*^ mice [11] and deletion of Ccr2 ameliorated retinal degeneration in another model [23]. These observations are consistent with the co-expression of Ccr2-RFP in amoeboid-shaped Cx3cr1-GFP-positive microglia in *Mertk*^*−/−*^*Cx3cr1*^*GFP/+*^*Ccr2*^*RFP/+*^ mice (Figs. 2, 3, and 4). Our results suggest that amoeboid-shaped activated microglia, which express both Cx3cr1 and Ccr2, are the dominant cell type in the subretinal space where they are activated by dead photoreceptors. Thus, Ccr2 expression in microglia is a feature of their activation during retinal degeneration. *Mertk*^*−/−*^*Cx3cr1*^*GFP/+*^*Ccr2*^*RFP/+*^ mice enabled us to observe the switch of microglial phenotype from resting to activated state by monitoring Ccr2-RFP expression in vivo. However, other chemokines and chemokine receptors should also be tested. Previously, we reported an early peak of MIP-1 chemokines including C-C motif ligand (CCL)3 and CCL4 (compared to CCL2 or other chemokines) in light-induced *Abca4*^*−/−*^*Rdh8*^*−/−*^ mice, which show dramatic PCD [11]. The *Mertk*^*−/−*^*Cx3cr1*^*GFP/+*^*Ccr2*^*RFP/+*^ mice described here will be useful to develop future microglia-targeted treatment strategies for retinal degeneration.

## Conclusions

Newly developed *Mertk*^*−/−*^*Cx3cr1*^*GFP/+*^*Ccr2*^*RFP/+*^ mice were used to monitor the migration of Cx3cr1-GFP-positive microglia from the inner retina to the outer retina and subretinal space. Round-shaped Ccr2-RFP-positive monocyte-derived macrophages also invaded the retina. Activated microglia and macrophages that had migrated to the OS layer and subretinal space were Cx3cr1-GFP and Ccr2-RFP dual positive. Initiation of CCR2 expression in CX3CR1-positive microglia is a feature of microglial activation in RD. Currently, microglia suppressive approaches are being evaluated as new treatment strategies for retinal diseases including AMD, RP, and diabetic macular edema [3, 24, 25]. *Mertk*^*−/−*^*Cx3cr1*^*GFP/+*^*Ccr2*^*RFP/+*^ mice will be a suitable model to assist in the development of future microglia-targeted treatment strategies.

## Abbreviations

CCL: C-C motif ligand
CNS: central nervous system
EAE: experimental autoimmune encephalomyelitis
IHC: immunohistochemistry
IS: inner segments
*Mertk*: c-mer proto-oncogene tyrosine kinase
ONL: outer nuclear layer
OS: outer segments
PCD: photoreceptor cell death
RCS: Royal College of Surgeons
RD: retinal degeneration
WBC: white blood cells
